# Supplementary material for: Proteome profiling of home-sampled dried blood spots reveals proteins of SARS-CoV-2 infections
Source: Commun Med (Lond). 2024 Apr 2;4:55. doi: 10.1038/s43856-024-00480-4 (PMC10987641; doi:10.1038/s43856-024-00480-4)
Supplement: Supplementary file 2 — Supplementary Information [file 43856_2024_480_MOESM2_ESM.pdf]

Supplementary Materials for

Proteome profiling of home-sampled dried blood spots reveals proteins of SARS-CoV-2 infections.

Claudia Fredolini, Tea Dodig-Crnković, Annika Bendes, Leo Dahl, Matilda Dale, Vincent Albrecht, Cecilia Mattsson, Cecilia E Thomas, Åsa Torinsson Naluai, Magnus Gisslen, Olof Beck, Niclas Roxhed* and Jochen M Schwenk*

*Corresponding authors' emails:
jochen.schwenk@scilifelab.se and roxhed@kth.se

This PDF file includes:

Figures: Supplementary Fig. 1 to 5

Tables: Supplementary Tab. 1 and 2

Supplementary Fig. 1. Global analysis of sample sets and protein panels. PCA, UMAP, and median-IQR analyses were performed on **(a)** study 1, **(b)** study 2, and **(c)** study 3 for each protein panel separately. The color codes indicate the serostatus of the groups compared within each study set.

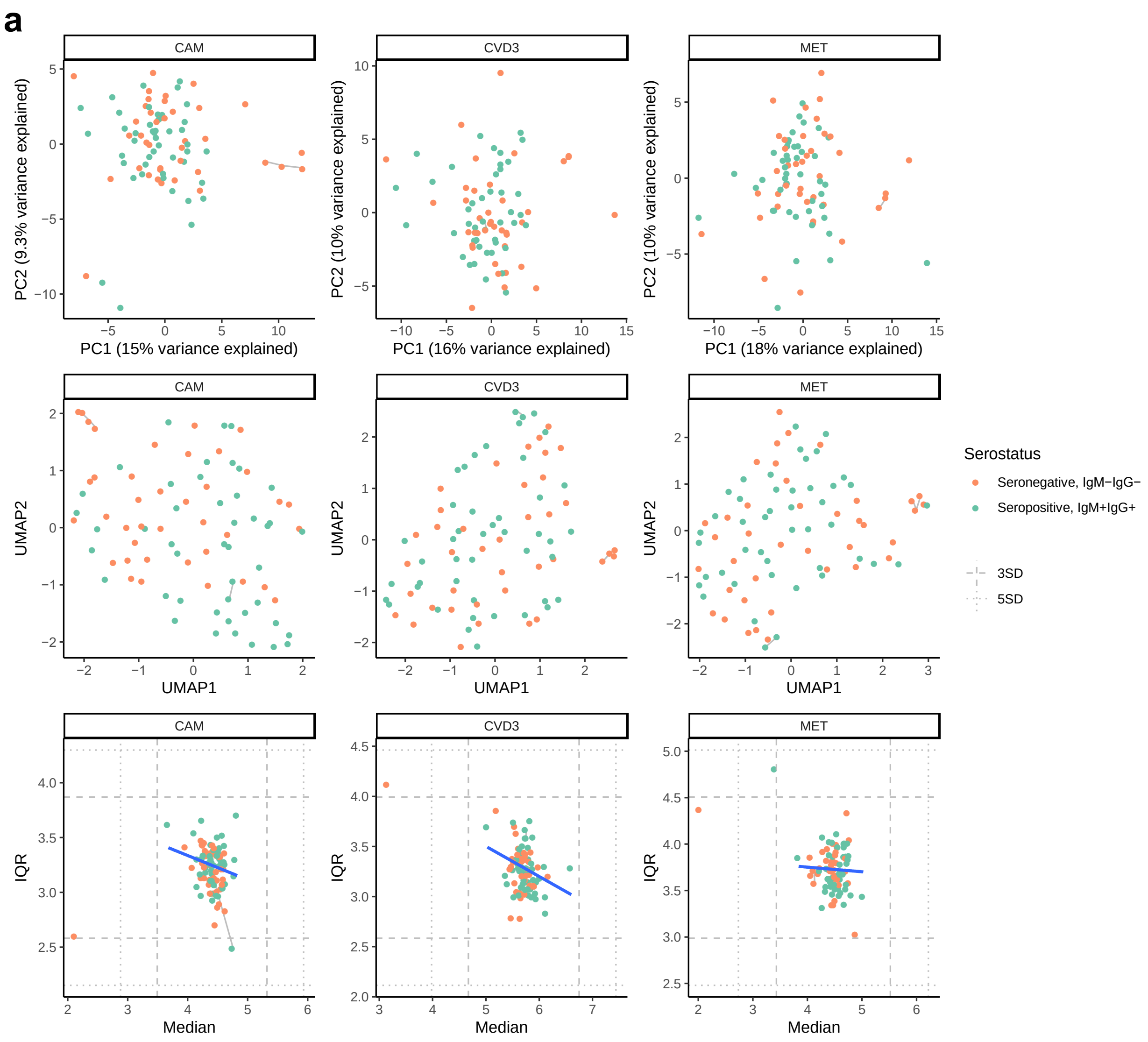

b

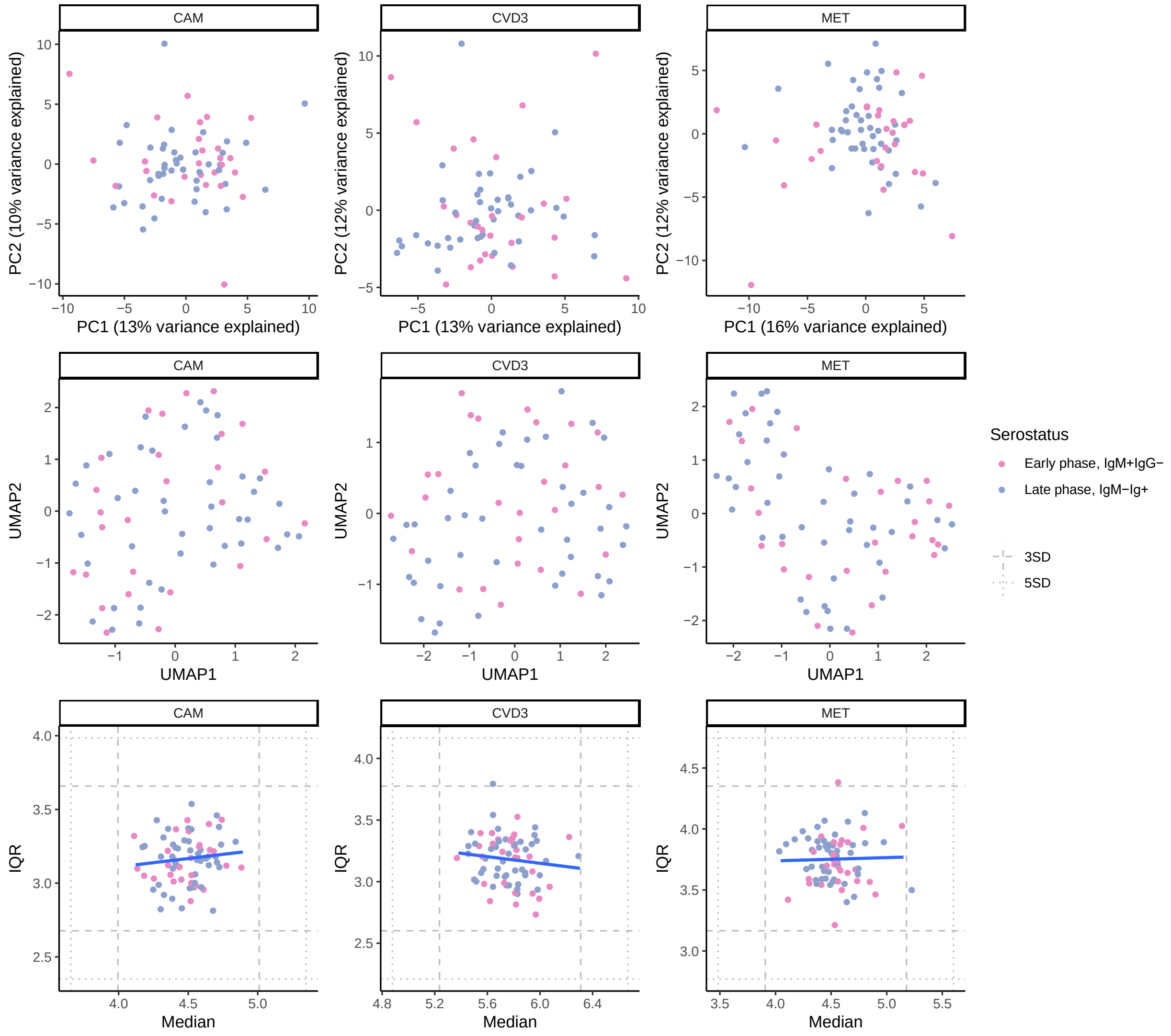
CAM
CVD3
MET
PC2 (10% variance explained)
PC2 (12% variance explained)
PC1 (13% variance explained)
PC1 (16% variance explained)
UMAP2
UMAP1
IQR
Median
Serostatus
Early phase, IgM+IgG−
Late phase, IgM−Ig+
3SD
5SD

c

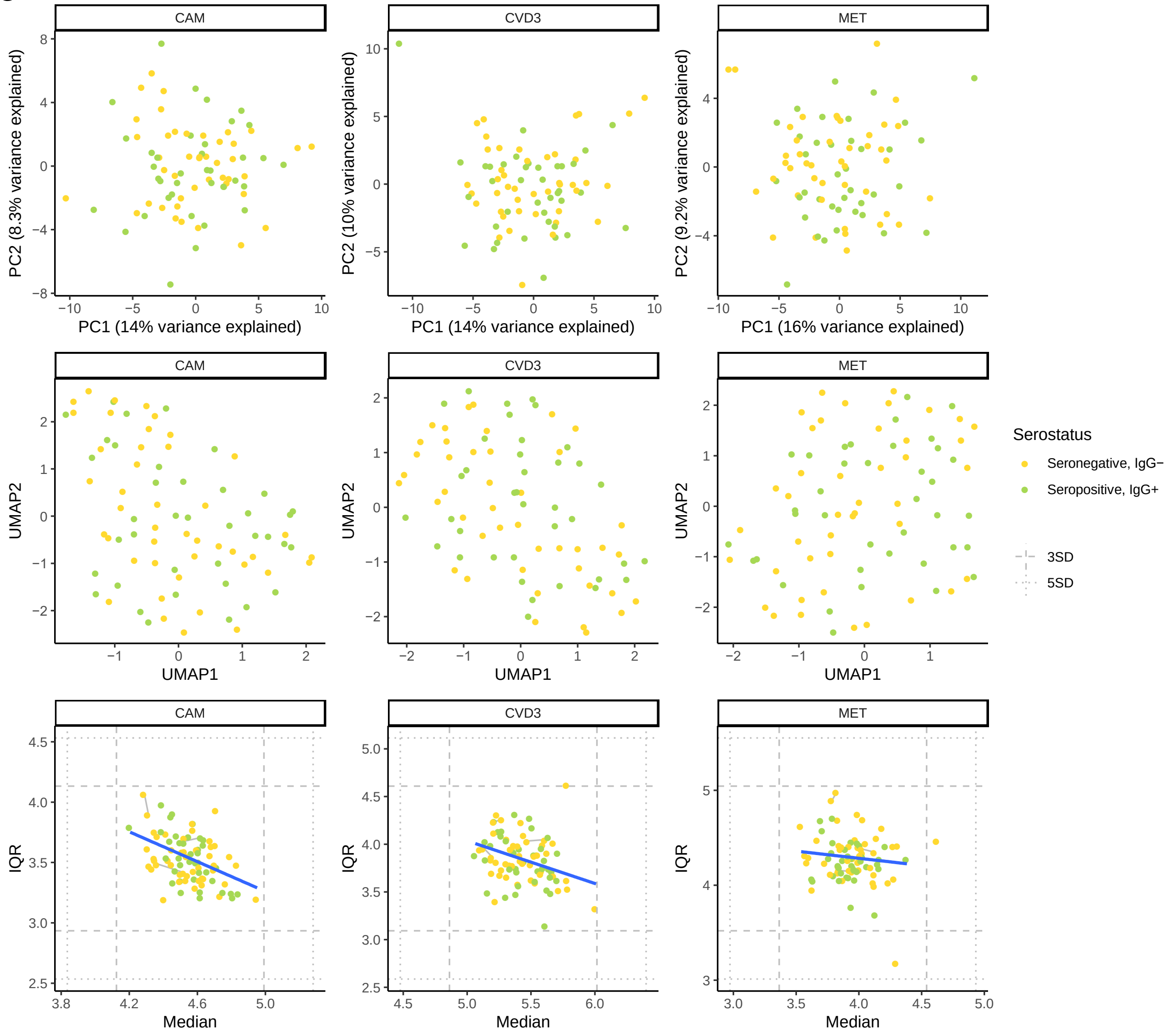

CAM
CVD3
MET
PC2 (8.3% variance explained)
PC1 (14% variance explained)
PC2 (10% variance explained)
PC1 (14% variance explained)
PC2 (9.2% variance explained)
PC1 (16% variance explained)
UMAP2
UMAP1
Serostatus
Seronegative, IgG−
Seropositive, IgG+
3SD
5SD
IQR
Median

Supplementary Fig. 2. Overview of protein-protein correlations. The distribution of protein-protein correlations across the sample and study sets center new zero.

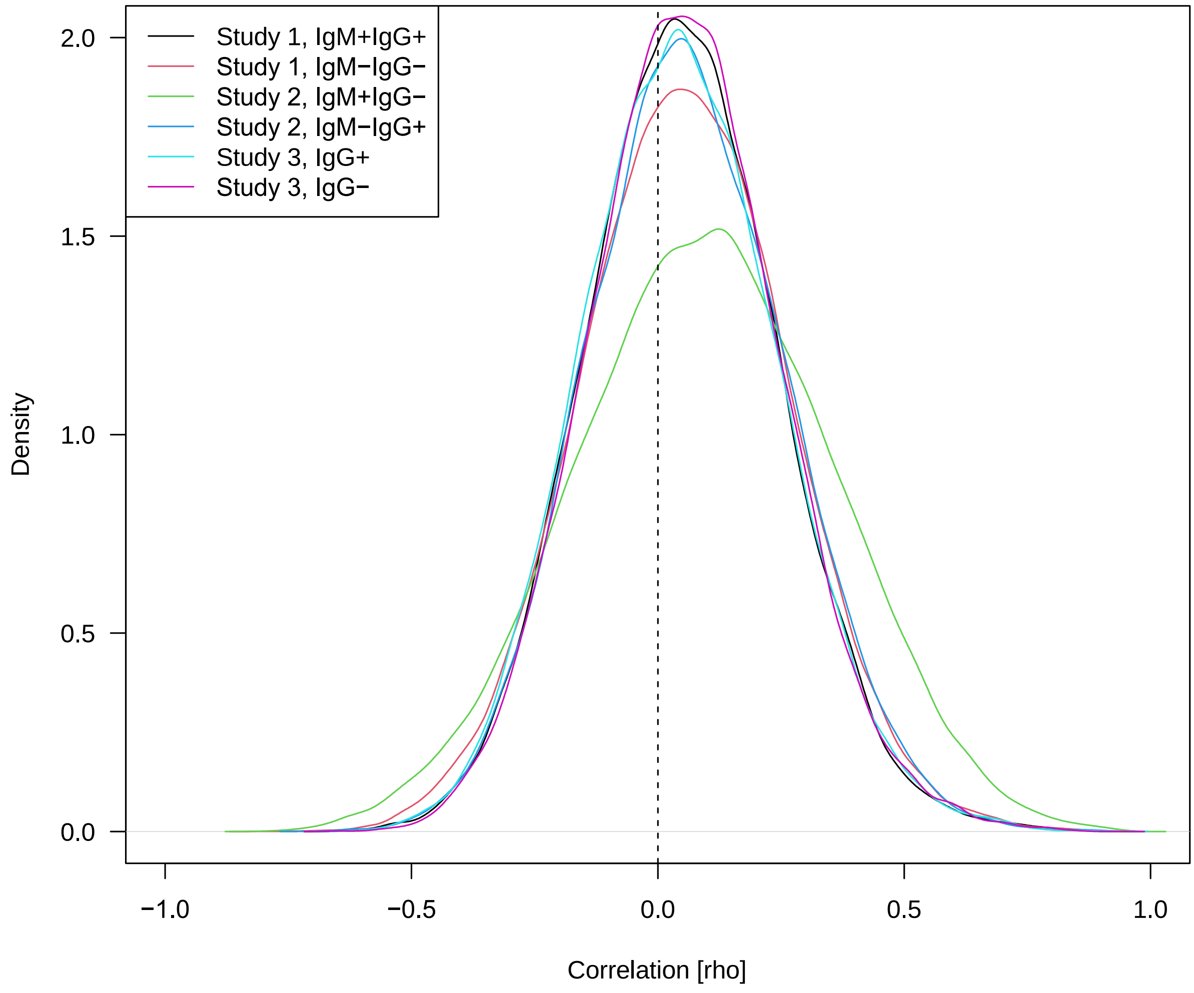

Supplementary Fig. 3. Direction of association. Concordance of the beta values obtained from the logistic regression analysis for **(a)** age and **(b)** sex in the three study sets. Only associations with p-value < 0.05 are included.

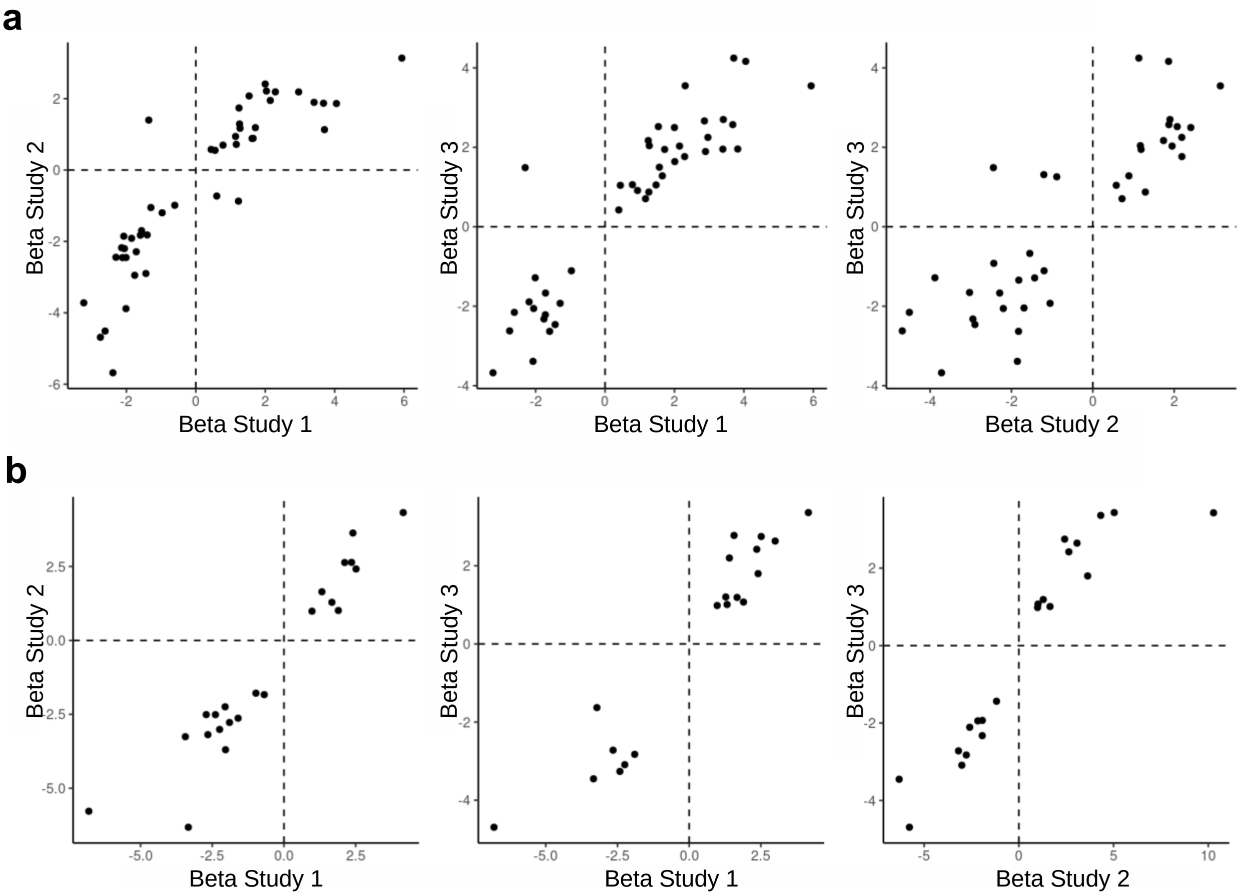

Supplementary Fig. 4. Volcano plots of correlations between protein levels and three serological markers. Each column represents the antibodies detected against SARS-CoV-2 antigens (N, RBD, and S). Each row represents the results for **(a)** study 1, **(b)** study 2, and **(c)** study 3.

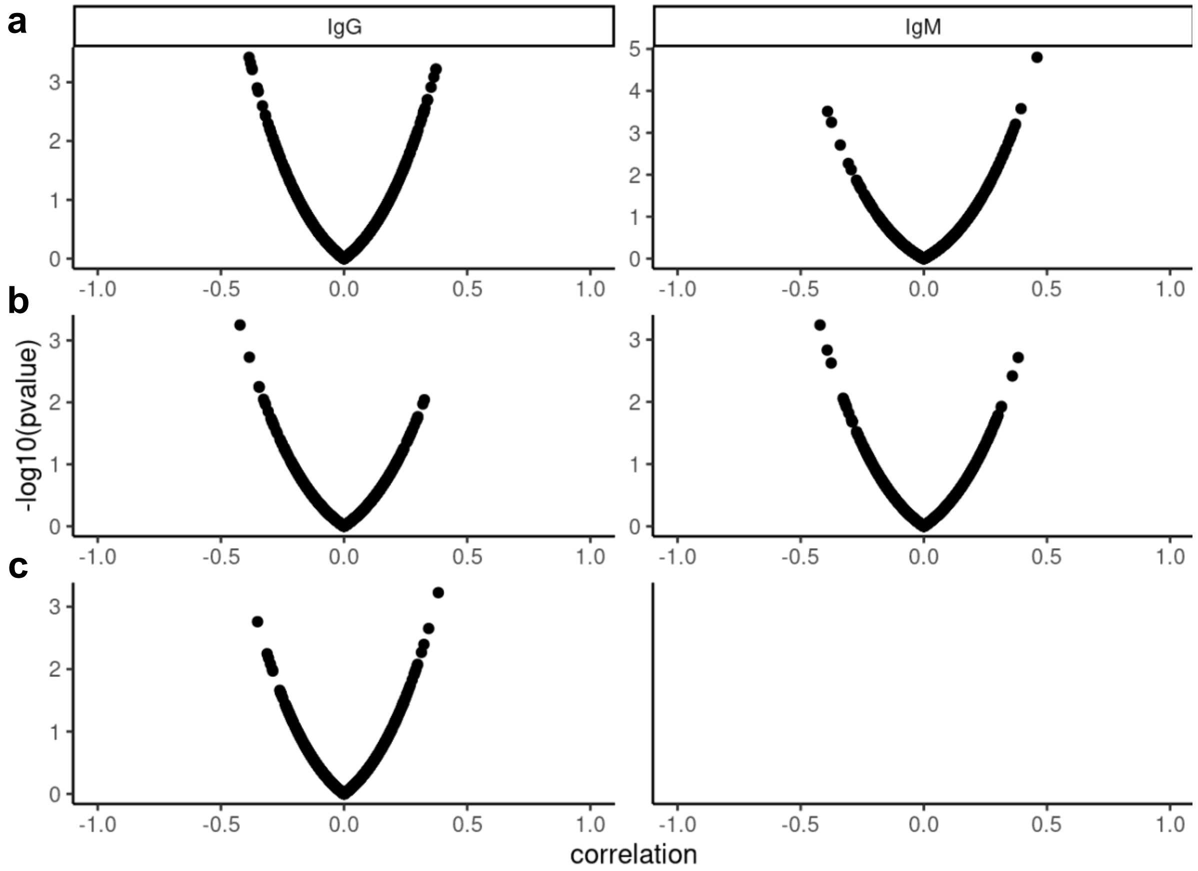

Supplementary Fig. 5. Longitudinal profiles from single donor post-infection. We performed SOTA clustering using nine groups to identify distinguishable protein profiles. Shown are the representative average protein profiles by group. In cluster 1, ANXA4, MBL2, F11, or SDC4 are found. Increasing trends of cluster 5 include anti-S IgG and the protein ANG. Decreasing trends in cluster 9 include anti-S IgM, FCGR2A, LILRB1, CR2, or IL2RA. The y-axes show the z-scores.

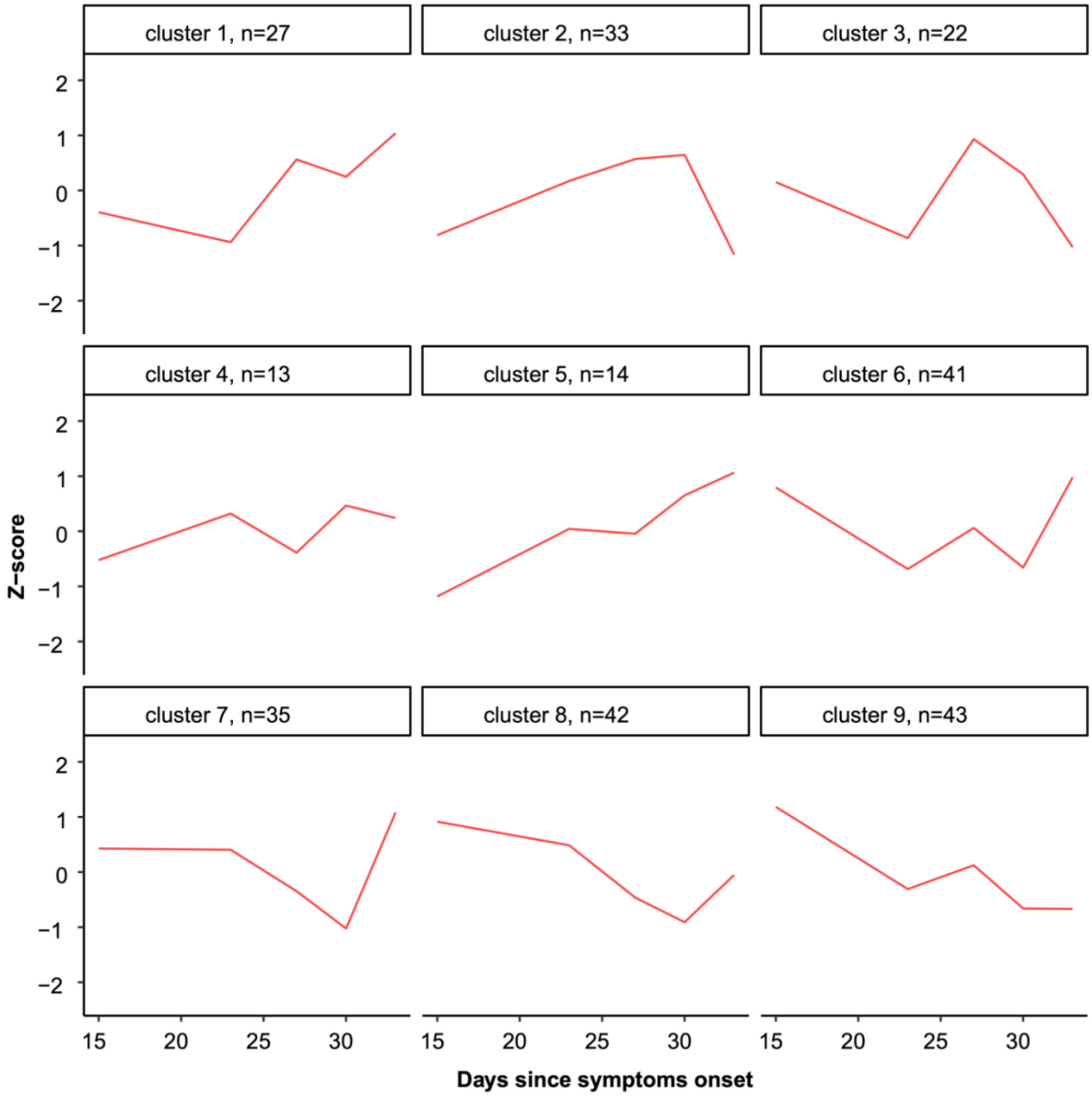

Supplementary Tab. 1. DBS versus plasma. Annotation of blood cell RNA expression categories.

Blood Cell RNA	N	Mean ΔNPX	SD ΔNPX	Mean rho	SD rho
Not detected	28	-0.02	0.87	0.80	0.20
Detected in many	23	0.37	1.23	0.72	0.26
Detected in some	17	0.72	1.51	0.63	0.30
Detected in all	14	2.01	2.61	0.45	0.42
Detected in single	10	1.35	2.48	0.55	0.37

SD = standard deviation

Supplementary Tab. 2. DBS versus plasma. Annotation of protein secretion categories.

Location of secretion	N	Mean ΔNPX	SD ΔNPX	Mean rho	SD rho
Not secreted (leakage)	27	0.83	2.14	0.61	0.41
Other main location + Blood	24	1.30	2.08	0.61	0.28
Blood	20	0.26	0.96	0.71	0.26
Intracellular/Membrane	8	0.93	1.49	0.65	0.32
Matrix	6	-0.39	0.40	0.82	0.09
Other locally	4	-0.44	0.20	0.80	0.11
Gastric	2	-0.16	0.11	0.95	0.03
Secreted - no data	1	0.38	-	0.90	-

SD = standard deviation
