# Supplementary material for: Proteome profiling of home-sampled dried blood spots reveals proteins of SARS-CoV-2 infections
Source: Commun Med (Lond). 2024 Apr 2;4:55. doi: 10.1038/s43856-024-00480-4 (PMC10987641; doi:10.1038/s43856-024-00480-4)
Supplement: Supplementary file 3 — Description of Additional Supplementary Files [file 43856_2024_480_MOESM3_ESM.pdf]

Description of Additional Supplementary Files

File Name: Supplementary Data 1
Description: Comparison of protein levels between DBS and EDTA plasma samples.

File Name: Supplementary Data 2
Description: Inter-quartile ranges, ranks, and CVs of protein levels in the three studies.

File Name: Supplementary Data 3
Description: Cluster and protein-protein correlation analysis.

File Name: Supplementary Data 4
Description: Association of protein levels with assays, donor traits, and serostatus; protein median values per serostatus group; tests of heterogeneity of variance; and correlations between proteins and anti-SARS-CoV-2 antibodies.

File Name: Supplementary Data 5
Description: Source data
