# Supplementary material for: Proteome profiling of home-sampled dried blood spots reveals proteins of SARS-CoV-2 infections
Source: Commun Med (Lond). 2024 Apr 2;4:55. doi: 10.1038/s43856-024-00480-4 (PMC10987641; doi:10.1038/s43856-024-00480-4)
Supplement: Supplementary file 9 — Reporting summary [file 43856_2024_480_MOESM9_ESM.pdf]

nature portfolio

Corresponding author(s): Niclas Roxhed, Jochen Schwenk

Last updated by author(s): Mar 5, 2024

Reporting Summary

Nature Portfolio wishes to improve the reproducibility of the work that we publish. This form provides structure for consistency and transparency in reporting. For further information on Nature Portfolio policies, see our Editorial Policies and the Editorial Policy Checklist.

Statistics

For all statistical analyses, confirm that the following items are present in the figure legend, table legend, main text, or Methods section.

n/a	Confirmed	
☐	☒	The exact sample size (*n*) for each experimental group/condition, given as a discrete number and unit of measurement
☐	☒	A statement on whether measurements were taken from distinct samples or whether the same sample was measured repeatedly
☐	☒	The statistical test(s) used AND whether they are one- or two-sided<br>*Only common tests should be described solely by name; describe more complex techniques in the Methods section.*
☐	☒	A description of all covariates tested
☐	☒	A description of any assumptions or corrections, such as tests of normality and adjustment for multiple comparisons
☐	☒	A full description of the statistical parameters including central tendency (e.g. means) or other basic estimates (e.g. regression coefficient) AND variation (e.g. standard deviation) or associated estimates of uncertainty (e.g. confidence intervals)
☐	☒	For null hypothesis testing, the test statistic (e.g. *F*, *t*, *r*) with confidence intervals, effect sizes, degrees of freedom and *P* value noted<br>*Give P values as exact values whenever suitable.*
☒	☐	For Bayesian analysis, information on the choice of priors and Markov chain Monte Carlo settings
☒	☐	For hierarchical and complex designs, identification of the appropriate level for tests and full reporting of outcomes
☐	☒	Estimates of effect sizes (e.g. Cohen's *d*, Pearson's *r*), indicating how they were calculated

Our web collection on statistics for biologists contains articles on many of the points above.

Software and code

Policy information about availability of computer code

Data collection	Protein levels were reported as NPX values obtained from Olink PEA assays. Readout of the assays were done by quantifitative microfluidic real-time PCR (Biomark HD, Fluidigm). Data were processed using the software NPX Manager (v.2.1.0.224 and v2.2.0.288, Olink Proteomics AB) and processed as described.<br><br>Collection of serological data was conducted as described in Roxhed, N., Bendes, A., Dale, M. et al. Multianalyte serology in home-sampled blood enables an unbiased assessment of the immune response against SARS-CoV-2. Nat Commun 12, 3695 (2021). https://doi.org/10.1038/s41467-021-23893-4
Data analysis	All data analyses and visualizations were performed using R version 3.6.0. and all used R packages are described in the manuscript. Analysis codes used in the study are available at https://doi.org/10.5281/zenodo.10782254

For manuscripts utilizing custom algorithms or software that are central to the research but not yet described in published literature, software must be made available to editors and reviewers. We strongly encourage code deposition in a community repository (e.g. GitHub). See the Nature Portfolio guidelines for submitting code & software for further information.

Data

Policy information about availability of data

All manuscripts must include a data availability statement. This statement should provide the following information, where applicable:

- Accession codes, unique identifiers, or web links for publicly available datasets
- A description of any restrictions on data availability
- For clinical datasets or third party data, please ensure that the statement adheres to our policy

The proteomics and serology data supporting the findings in this study are available at the SciLifeLab Data Repository (https://scilifelab.figshare.com) under the doi identifiers 10.17044/scilifelab.2505042255 and 10.17044/scilifelab.1455552056. As described in the repository, access to the data can be granted for validation purposes and upon reasonable request to the corresponding authors. Source data are available as Supplementary Data 5.

Human research participants

Policy information about studies involving human research participants and Sex and Gender in Research.

Reporting on sex and gender	Information about sex was obtained from self-reported questionnaires and protein levels for their associations with this trait.
Population characteristics	Donors provided answers to the following questions (see Tables 1-3 for details in relation to serostatus)<br>Sex: Female, Male, Missing<br>Age groups: 20-29, 30-39, 40-49, 50-59, 60-69, 70-74, Missing<br>Influenza-like symptoms: Yes, severe; Yes, fever; Yes, mild; No; Missing<br>Breathing symptoms: Yes; Coughing; Difficulty breathing; Both; No; Missing<br>Sensing symptoms: Loss of taste; Loss or smell; both; None; Missing<br>Vaccination: Yes; No; Missing
Recruitment	We asked for addresses of random participants aiming to achieve an equal age and sex distribution of blood donors from the general public.<br><br>Individuals who volunteered to participate in the study were asked to perform self-sampling according to the instructions and return the filled sampling card, questionnaire, and consent form by regular mail. All cards were barcoded and stored at room temperature until use or as stated otherwise.<br><br>Capillary blood samples were obtained from the general population by cold-mailing home-sampling kits (MM20-009-01, Capitainer AB, Sweden).During April 2020, 2000 individuals (20-74 years old) in metropolitan Stockholm were mailed a sampling kit together with a questionnaire and as described previously (Roxhed et al, 2021, Nature Communications). In May 2021, kits and a questionnaire were sent to 2000 randomly selected individuals (18-70 years old) in metropolitan Stockholm and Gothenburg.
Ethics oversight	All blood donors gave informed, documented consent. The studies were approved by the regional ethical board (EPN Stockholm, Dnr 2015/867-31/1) and the Swedish Ethical Review Authority (EPM, Dnr 2020-01500 and 2021-01106).

Note that full information on the approval of the study protocol must also be provided in the manuscript.

Field-specific reporting

Please select the one below that is the best fit for your research. If you are not sure, read the appropriate sections before making your selection.

☒ Life sciences ☐ Behavioural & social sciences ☐ Ecological, evolutionary & environmental sciences

For a reference copy of the document with all sections, see nature.com/documents/nr-reporting-summary-flat.pdf

Life sciences study design

All studies must disclose on these points even when the disclosure is negative.

Sample size	Samples from 228 donors were selected based on previously obtained serological analysis.
Data exclusions	Outlier samples were identified by IQR and median values of NPX values per samples, see Supplementary Fig. 1. Those that exceeded 3x SD of the IQR or median values were flagged as outliers.
Replication	Our study replicated findings made from the analysis of clinically collected serum/plasma samples.<br>Replicated protein analysis of DBS samples revealed that > 90% of the protein assays had a CV < 10%.
Randomization	Serostatus was determined in a multi-analyte assay and used to assign donors into groups. Since not all the individuals experienced symptoms and could not track back the time of infection, we considered positivity for both IgM and IgG, or only for one immunoglobulin type.

In April 2020, as previously described in Roxhed et al 2021, sampling kits and a questionnaire were sent to 2000 randomly selected individuals (20-74 years old) in metropolitan Stockholm (Tables 1. and 2.). Only a specific subset of individuals with defined serostatus and matching self-reported symptoms were considered for this study.

In May 2021, kits and a questionnaire were sent to 2000 randomly selected individuals (18-70 years old) in metropolitan Stockholm and Gothenburg (Table 3). Only unvaccinated donors were considered for this study.

Blinding	Sample groups were defined before the analysis, hence information about the serostatus was not blinded. Samples were randomized within each study set.

Reporting for specific materials, systems and methods

We require information from authors about some types of materials, experimental systems and methods used in many studies. Here, indicate whether each material, system or method listed is relevant to your study. If you are not sure if a list item applies to your research, read the appropriate section before selecting a response.

Materials & experimental systems

n/a	Involved in the study
☐	☒ Antibodies
☒	☐ Eukaryotic cell lines
☒	☐ Palaeontology and archaeology
☒	☐ Animals and other organisms
☐	☒ Clinical data
☒	☐ Dual use research of concern

Methods

n/a	Involved in the study
☒	☐ ChIP-seq
☒	☐ Flow cytometry
☒	☐ MRI-based neuroimaging

Antibodies

Antibodies used	Antibodies used in this study were part of the Olink PEA assays.
Validation	Validation data is provided by Olink Proteomics AB: https://www.olink.com/our-platform/assay-validation

Clinical data

Policy information about clinical studies
All manuscripts should comply with the ICMJE guidelines for publication of clinical research and a completed CONSORT checklist must be included with all submissions.

Clinical trial registration	n/a
Study protocol	n/a
Data collection	n/a
Outcomes	n/a
